# Structure and Function of the Photosystem Supercomplexes

**DOI:** 10.3389/fpls.2018.00357

**Published:** 2018-03-20

**Authors:** Jinlan Gao, Hao Wang, Qipeng Yuan, Yue Feng

**Affiliations:** ^1^State Key Laboratory of Plant Genomics, Institute of Microbiology, Chinese Academy of Sciences, Beijing, China; ^2^College of Life Science and Technology, Beijing University of Chemical Technology, Beijing, China

**Keywords:** chloroplast, photosynthesis, structure, photosystem II protein complex, protein complex

## Abstract

Photosynthesis converts solar energy into chemical energy to sustain all life on earth by providing oxygen and food, and controlling the atmospheric carbon dioxide. During this process, the water-splitting and oxygen-evolving reaction is catalyzed by photosystem II (PSII), while photosystem I (PSI) generates the reducing power for the reduction of NADP^+^ to NADPH. Together with their peripheral light-harvesting complexes (LHCs), photosystems function as multisubunit supercomplexes located in the thylakoid membranes of cyanobacteria, algae, and plants. Recent advances in single-particle cryo-electron microscopy (cryoEM), X-ray free electron laser (XFEL) and other techniques have revealed unprecedented structural and catalytic details concerning the two supercomplexes. Several high-resolution structures of the complexes from plants were solved, and serial time-resolved crystallography and “radiation-damage-free” femtosecond XFEL also provided important insights into the mechanism of water oxidation. Here, we review these exciting advances in the studies of the photosystem supercomplexes with an emphasis on PSII-LHCII, propose presently unresolved problems in this field, and suggest potential tendencies for future studies.

## Introduction

Photosynthesis carries out a series of biophysical and biochemical processes, finally converting solar energy into chemical energy. Oxygenic photosynthesis split water molecules to oxygen, which is indispensable for maintaining aerobic life on earth (Dismukes et al., 2001). It is believed that photosynthesis has evolved only once during the evolution history in cyanobacteria. For algae and higher plants, they acquired photosynthesis capacity via cyanobacteria endosymbionts which evolved to chloroplasts in plants (Tomioka and Sugiura, 1983). Plant leaves are the major organs of photosynthesis with about 100 chloroplasts in each mesophyll cell (Woodson, 2016). Due to their essential roles in light harvesting and energy production, chloroplasts are vital organelles of photosynthetic cells in algae and higher plants, acting as the suppliers of carbon sources and energy. Chloroplast is a large organelle with a complex structure harboring two outer membranes called the chloroplast envelope and a third extensively folded internal membrane system called thylakoid (Arvidsson and Sundby, 1999; Kirchhoff, 2013). The thylakoid membrane is composed of two morphologically distinct domains, the grana domain which is characterized by ∼5–20 layers of cylindrical stacks of thylakoid membrane disks (Mullineaux, 2005; Mustárdy et al., 2008), and the stroma lamellae domain which are stroma-exposed membrane pairs connecting the grana stacks (Dekker and Boekema, 2005).

There are two types of photosystems in cyanobacteria, algae and higher plants, called photosystem I (PSI, plastocyanin-ferredoxin oxidoreductase) and photosystem II (PSII, water-plastoquinone oxidoreductase), both of which are multisubunit membrane complexes. The PSI is located in the stroma lamella of thylakoid while the PSII is in the stacked grana domain (Albertsson, 2001; Dekker and Boekema, 2005). Each photosystem is composed of a core complex and a peripheral antenna system, light harvesting complex I (LHCI) for PSI and light harvesting complex II (LHCII) for PSII, respectively. Recently, new atom-resolution structures of the photosystems and detailed insights into the water-splitting process have been reported, with the development of single-particle cryo-electron microscopy, serial time-resolved crystallography and other techniques. Here, we will review the recent progress in the studies into the structures and functions of photosystems of different origins, with an emphasis on PSII-LHCII.

## Biochemistry of Photosystems

The complete photosynthetic reactions in cyanobacteria, algae and plants are executed by four major protein supercomplexes including PSI, PSII, cytochrome b_6_f (plastoquinone-plastocyanin oxidoreductase) and F-ATPase (proton-motive force-driven ATP synthase) (Nelson and Benshem, 2004; Nelson and Yocum, 2006). Both PSI and PSII supercomplexes bind chlorophyll molecules to sense different spectrums and intensities of light (Remelli et al., 1999; Nelson and Yocum, 2006; Croce and van Amerongen, 2013; van Amerongen and Croce, 2013; Caffarri et al., 2014; Ruban, 2015). Light harvested by the chlorophylls and other pigments in PSI and PSII is transferred to the photosynthetic reaction center (RC), further inducing the excitation of chlorophylls known as P680 for PSII and P700 for PSI to initiate the proton translocation across the membrane (Nelson and Junge, 2015). In PSII, P680 undergoes charge separation and the generated electrons are transferred to the quinone acceptor pheophytin and plastoquinone sequentially (Grabolle and Dau, 2005; Johnson, 2016). Meanwhile, water molecule, the authentic electron donor, is oxidized to molecular oxygen and P680 is eventually reduced. After the reaction, the electrons are ultimately transferred to the thylakoid-embedded cytochrome b_6_f, which oxidizes plastoquinols to plastoquinones and reduces plastocyanins (Cramer et al., 1996). And then, the plastocyanin is oxidized by PSI, during which the reduced electron carrier protein ferredoxin, is used to reduce NADP^+^ to NADPH by ferredoxin–NADP^+^ reductase (FNR) enzyme (Brettel and Leibl, 2001; Sétif, 2001; Golbeck, 2006). Together, PSII generates the most positive redox potential, while PSI generates the powerful naturally occurring reductant NADPH (Hope, 2000; Holzwarth et al., 2006; Nelson, 2011). The photocatalytic activity of PSII and PSI is linked by the cytochrome b_6_f complex, and the proton-motive force generated during the process are utilized by the F-ATPase to generate ATP, which together with NADPH are supplied as energy compounds for sugar synthesis from carbon dioxide by the dark reaction (Pfannschmidt, 2003).

The first PSI structure from the thermophilic cyanobacterium *Synechococcus elongatus* (*S. elongatus*) displayed a complex with 12 protein subunits and 127 cofactors (96 chlorophylls, 22 carotenoids, 2 phylloquinones, 3 Fe_4_S_4_ clusters, and 4 lipids), providing the very first detailed molecular architecture of PSI (Fromme et al., 2001; Jordan et al., 2001). *S. elongatus* PSI is a trimer with a diameter of 210 Å and a maximum height of 90 Å (Jordan et al., 2001), while the plant PSI supercomplex is a monomer (Ben-Shem et al., 2003). The core complex is largely conserved from cyanobacteria to plants with nine membrane-embedded subunits, whereas the LHCI complexes are variable in subunit composition, binding pigments and sizes due to the different habitats of cyanobacteria, algae, and plants (Ben-Shem et al., 2003; Qin et al., 2015). However, although PSI and PSII evolved from the same ancestor, belonging to the same superfamily, their structures are largely different.

## Structure of the Cyanobacterial PSII Supercomplex

**FIGURE 1:**
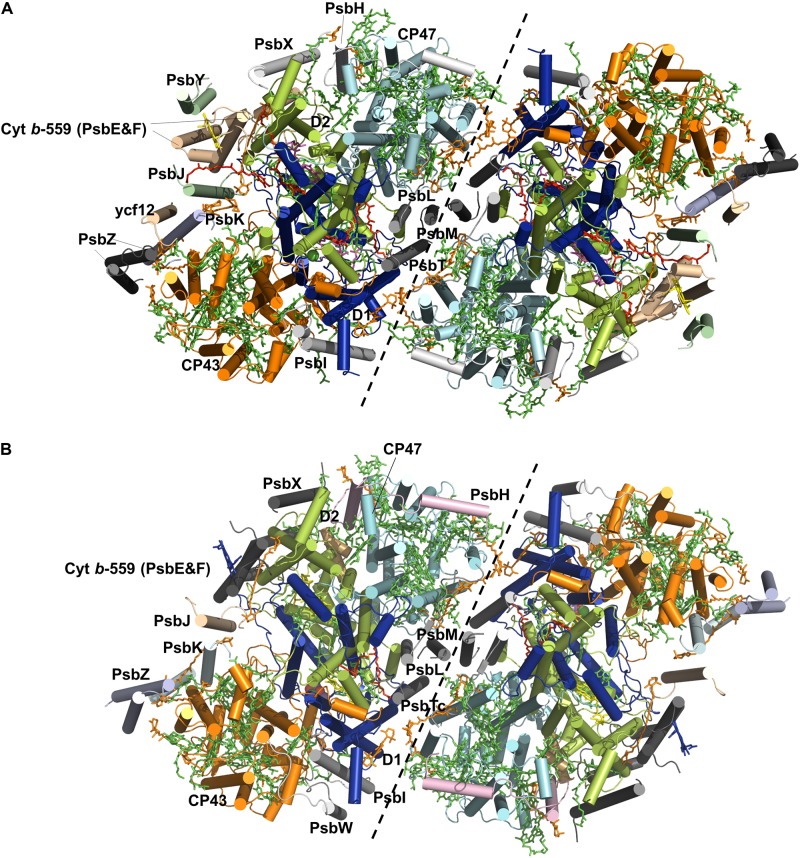
Overall structures of the PSII core complex from *Tynechococcus elongatus* **(A)** and spinach **(B)**. The monomer-monomer interface is indicated by a black dashed line. Only the four large subunits and the intrinsic subunits of PSIIs are shown. The subunits D1 (blue), D2 (lime), CP43 (yellow), and CP47 (cyan) and the other small subunits are labeled in the monomer on the left. Cofactors are shown in sticks, including Chl (green), Car (orange), heme (light magenta) and lipids (red). The Mn cluster is shown in spheres.

The PS II homodimer from *Thermosynechococcus elongatus* (*T. elongatus*) has dimensions of 105 Å in depth (45 Å in membrane), 205 Å in length, and 110 Å in width (Ferreira et al., 2004). The PSII supercomplex in cyanobacteria comprises the reaction center (RC) proteins D1 and D2, the antenna subunits CP47 and CP43, 13 membrane-intrinsic small subunits (PsbE, PsbF, PsbH-M, PsbN, PsbX, PsbY, PsbZ, and PsbYcf12) and 3 extrinsic subunits (PsbO, PsbU, and PsbV). The structures of D1 and D2 are similar to each other, both containing five helices all tilted against the membrane planes (Zouni et al., 2001; Kamiya and Shen, 2003), and they form the center of the PSII complex. CP43 and CP47 surround the D1-D2 core with similar structures of six helices, respectively. Afterward, Loll et al. (2005) provided the first complete structure of cyanobacterial photosystem II, providing a full glimpse of the PSII cofactors. They displayed the positions of 20 protein subunits and their interactions with 77 cofactors (**Figure 1A**). The overall structures of the supercomplex and protein subunits are similar to those previously reported (Zouni et al., 2001; Kamiya and Shen, 2003; Biesiadka et al., 2004). Lipids have long been thought to play a role in the assembly and function of PSII, and for the first time the authors showed the lipid integrally bound to PSII. Eleven lipids surrounding the RC form a belt to separate it from the antenna and small protein subunits, while the remaining lipids are mostly located at the monomer-monomer interface. The lipid-rich property renders PSII both structural flexibility for local mobility and convenience in subunit-subunit recognition (Guskov et al., 2009). Eleven carotenoid molecules were modeled as β-carotenes in all-trans configurations in their study and an additional Car15 was identified in the study by Guskov et al. (2009). In the study by Guskov et al. (2009), they also successfully assigned the small protein subunits Psbycf12, PsbY, and PsbX to the previously unassigned positions (Loll et al., 2005; Guskov et al., 2009). A summary of the subunit composition information, including the subunit-cofactor interactions in PSII from *T. elongatus*, is presented in **Table 1** (Guskov et al., 2009). In 2009, Broser et al. presented the first structure of a monomeric form of PSII core complex (PSIIcc) with high oxygen-evolution capacity from *T. elongatus* (Broser et al., 2010). The assembly of the protein subunits, tetrapyrrole cofactors and the non-heme iron in the monomeric PSIIcc are all identical to those in the dimer structure.

**Table 1:** Transmembrane (TM) helices, chlorophylls, and other cofactors of PSII RCs from cyanobacteria and plants^1^.

Subunits	TM helices		Chlorophylls		Other cofactors in plant PSII RC			
			
	Cyano	Plant	Cyano	Plant	Carotenoid	Heme	Lipid	Others
A (D1)	5	5	4	6^2^	1		3	1 Mn_4_CaO_5_
B (CP47)	6	6	16	16	3		2	
C (CP43)	6	6	13	13	3		4	
D (D2)	5	5	4	2	1		4	1 plastoquinone
E	1	1				1^3^		
F	1	1						
H	1	1			1		1	
I	1	1						
J	1	1						
K	1	1			1			
L	1	1					1	
M	1	1						
O	0	0						
P		0						
Q		0						
T	1							
U	0							
V	0							
W		1						
Tc		1						
Tn		0						
X	1	1						
Y	1							
Z	1	2					1	
Ycf12	1							


*^*1*^The subunit composition and the number of TM helices are based on the structures of PSIIs from *Tynechococcus elongatus* and spinach (Guskov et al., 2009; Wei et al., 2016). ^*2*^These include 4 Chl a and 2 pheophytin. ^*3*^This heme is bound by both PsbE and PsbF.*

During photosynthesis, water oxidation happens in the oxygen-evolving complex (OEC), which comprises the Mn_4_CaO_5_ cluster as the catalytic center. Water splitting is a process fulfilled in five consecutive stages named S_0_ to S_4_. It has been a model system for synthesizing catalysts for inorganic water oxidation and dioxygen evolution (Kanady and Agapie, 2011; Mukherjee et al., 2012). In the work of Ferreira et al. (2004), they reported that the OEC harbors a “cubane-like” Mn_3_CaO_4_ cluster linked to a fourth Mn by a mono-μ-oxo bridge which had not been specifically suggested before. However, neither water nor hydroxide could be observed to find the water oxidation site accurately in their study. In the first complete PSII structure, the Mn_4_Ca cluster was proposed as a “Y-shaped hook,” considerably differed from the “cubane-like” model (Loll et al., 2005). Then a 1.9-Å resolution X-ray structure of PSII from *T. vulcanus* revealed a clear picture of the Mn_4_CaO_5_ cluster, in which the electron densities for each metal ion and the oxo-bridged oxygen atoms were totally separated, thus allowing the clear assignment of each of the atoms. They found that the OEC Mn_4_CaO_5_ cluster displayed a “distorted chair” conformation with three Mn, one Ca and four oxygen atoms forming an asymmetric cubane-like seat base and the fourth Mn (Mn_4_) together with the fifth oxygen atom (O_4_) forming the chair back (Umena et al., 2011). Subsequently, a simultaneous femtosecond X-ray spectroscopy and diffraction of the PSII system showed that the electron density maps of the dark and illuminated states are similar with an overall correlation coefficient (CC) of 0.77 (a CC of 0 means no correlation; a CC of 1 indicates full correlation), suggesting no significant conformational changes between the S_1_ and S_2_ states (Kern et al., 2013). However, with a serial time-resolved crystallography, the authors acquired PSII structures in the dark S_1_ and putative S_3_ states, in which they found that the distance between the Mn_3_O_x_Ca cubane and the distant protruding Mn (dangler Mn) increased in the putative S_3_ state, allowing the binding of the second water molecule during the S_2_ to S_3_ state transition (Kupitz et al., 2014). Moreover, with a “radiation-damage-free” femtosecond X-ray free electron laser (XFEL), Suga et al. (2015, 2017) found that the Mn-Mn and Mn-O distances showed marked differences in the OEC from XFEL: all the distances are about 0.1–0.2 Å shorter than those from the X-ray diffraction (XRD) structures. In addition, the position of O5 is also unusual. The results showed that it functions more as a hydroxide ion instead of a normal oxygen dianion to serve as one of the substrate oxygen atoms (Suga et al., 2015). Their recent work described the light-induced structural changes in PSII by two-flash illumination, modeled a sixth oxygen atom (O6) close to O5, and provided important implications for the O=O bond formation mechanism (Suga et al., 2017). Furthermore, it was reported that the chloride ion is essential for oxygen evolution, and there are two anion binding sites positioned on the two sides of the MnCa cluster with the same distance from the cluster to stabilize its structure (Kawakami et al., 2009).

## Structure of the Plant PSII Supercomplex

Plant PSII has a similar overall structure of a dimeric supercomplex as cyanobacterial PSII. The first structure of plant PSII-LHCII complex from spinach was obtained 17 years ago at 17 Å (Nield et al., 2000), and recently a 3.2 Å spinach C_2_S_2_-type (C: PSII core complex; S: strongly associated LHCII trimer) supercomplex structure was reported with the development of single-particle cryo-electron microscopy techniques (Wei et al., 2016) (**Figure 1B**). More recently, structure of the dominant type of supercomplex in plants, the C_2_S_2_M_2_-type (M: moderately bound LHCIIs) was also solved at 2.7 and 3.2 Å for the stacked and unstacked forms from *Pisum sativum* (pea), and at 5.3 Å from *Arabidopsis thaliana*, respectively (Su et al., 2017; van Bezouwen et al., 2017). Compared to the structure of the spinach C_2_S_2_-type supercomplex, the structure reported by Su et al. (2017) was determined under more physiological conditions, containing three light-harvesting complex (LHC) monomers (CP29, CP26, and CP24) and two trimers (S-LHCII and M-LHCII) per core. In contrast, CP24 and M-LHCII are missing in the previous structure of Wei et al. (2016). The plant PSII indeed exhibits the same composition and organizations of the subunits and cofactors as their cyanobacterial counterparts (**Table 1**). The catalytic center within the core complex is composed of four largest membrane intrinsic subunits PsbA (D1), PsbB (CP47), PsbC (CP43), and PsbD (D2). Specifically, D1 and D2 form the photochemical RC, which is responsible for the charge separation and electron transfer, and CP47 and CP43 act as internal antenna proteins involved in light harvesting and energy transportation from peripheral antenna to the RC. In the core complex, there are also 12 low molecular-mass (MM) membrane-spanning subunits surrounding the reaction center, forming a belt-like structure. In the spinach PSII-LHCII complex, these subunits are PsbE, PsbF, PsbH-M, PsbTc, PsbW, PsbX, and PsbZ. Most of these subunits are structurally conserved with a single transmembrane helix except PsbZ with two helices. These subunits are essential for both the dimerization and stabilization of the core complex and the association between the core complex and the peripheral antenna complex. In addition, they bind cytochrome *b*-559 to protect the PSII complex from photo-damage. Three extrinsic subunits PsbO, PsbP, and PsbQ constitute the OEC, which also encompasses the luminal domain of CP43 and the C-terminal domain of D1, shielding the water splitting machinery. Among them, PsbO stabilizes the Mn complex while PsbP and PsbQ are involved in optimizing the oxygen evolution at physical concentration of calcium and chloride ions. Structure comparison also revealed that the flexible regions of these subunits experience significant conformational changes when they bind to the core complex (Wei et al., 2016). Outside the core complex is the LHCII, the structure of which in the PSII supercomplex is almost the same as the pea LHCII complex (Standfuss et al., 2005).

Plant LHCII occupies about 30% of total proteins in the chloroplast membrane, therefore representing the most abundant membrane protein on earth (Peter and Thornber, 1991; Standfuss et al., 2005). LHCII acts as a heterotrimer constituted by Lhcb1, Lhcb2, and Lhcb3. Each polypeptide spans the thylakoid membrane three times with its C terminus positioned on the luminal side (Kuhlbrandt et al., 1994). The LHCII complex is vital for both photosynthesis and chloroplast grana formation. For the first function, the LHCII heterotrimers are linked to the photosystem core complex by the minor antenna subunits Lhcb4 (CP29), Lhcb5 (CP26), and Lhcb6 (CP24). In spinach PSII-LHCII supercomplex, two LHCII heterotrimer together with two CP26 proteins flank the core dimer complex from both sides (Wei et al., 2016). Whereas in the C_2_S_2_M_2_ supercomplex from *Arabidopsis* and pea, there are four LHCII trimers, out of which the two strongly bound LHCII trimers (S_2_) along with CP26 and CP24, and the moderately bound trimers (M_2_) with CP29, together encompass the core complex for electron transportation (Su et al., 2017; van Bezouwen et al., 2017). For the second function, the stromal surface of the LHCII trimer is negatively charged whereas its N-terminal first 15 residues contain 4 positively charged residues. This striking charge pattern resembles a “Velcro-like” mode, guaranteeing non-specific interactions of LHCII trimers in the adjacent thylakoid membranes (Standfuss et al., 2005), which seems essential for the chloroplast grana formation. It has been reported that constitutively expression of Lhcb1 robustly increased grana stacks in the transgenic tobacco plants (Labate et al., 2004), while knock-down of Lhcb1 and Lhcb2 impede formation of grana stacks (Andersson et al., 2003; Garab, 2014, 2016). In a recent work, in order to figure out how PSII-LHCII supercomplexes interact with each other in the chloroplast thylakoid, the authors isolated the PSII-LHCII supercomplexes in an ionic concentration that resembles the chloroplast native environment, and found that most of the supercomplexes are existed in a paired C_2_S_2_M form (Albanese et al., 2017). This study provided new insights into how adjacent thylakoids might be linked to mediate the stacking of grana membranes by interactions between pairs of PSII-LHCII supercomplexes.

Cofactors within the PSII-LHCII supercomplex are indispensable for their appropriate functions. Similar to those in cyanobacteria, these cofactors mainly include chlorophylls, carotenoids, lipids etc. (**Table 1**). In spinach C_2_S_2_ PSII-LHCII supercomplex, there are in total 105 chlorophyll molecules, 28 β-carotenes and xanthophylls, one heme, one Mn_4_CaO_5_ cluster, one plastoquinone and numerous lipids. Interestingly, the LHCII monomer shows both amino acid sequence and structure similarities to those of CP29, however, the type, quantity and location of the chlorophylls they bind are significantly different (Liu et al., 2004; Standfuss et al., 2005; Pan et al., 2011). As the largest chromophore-bound antenna subunits, the LHCII harbors Chls which absorb solar radiation of different wavelengths of 660 ± 20 nm.

## Conclusions and Perspectives

Photosynthesis plays very important roles in molecular oxygen production, atmospheric carbon dioxide control and global food supply. Structural information of the photosystems is invaluable for our understanding of photosynthesis, probably the most important process on earth. The information will also help design artificial photosynthetic system for the improvement of bioenergy production and the enhancement of agricultural productivity. Most recently, the structure of the largest light-harvesting complex, the phycobilisome (PBS) from *Griffithsia pacifica* was also reported (Zhang et al., 2017). As the main light-harvesting antenna in cyanobacteria and red algae, it exhibits a very fast energy transfer rate with a high quantum yield (Glazer, 1989). The structural information of the PBS will provide a firm basis for understanding its energy transfer pathways and further applications in the designs of artificial light-harvesting machineries.

Recent advances in single-particle cryo-EM have provided unprecedented structural information about these huge membrane complexes. However, there are also several open questions to be answered. First, the exact reaction mechanism underlying water oxidation and possible structural rearrangements during the S-state transitions still await the structures of PSII in more intermediate S states. Second, it is still not well understood why in PSII only one electron transfer chain is functional (as in bacterial RC), whereas in PSI both are functional (Santabarbara et al., 2010). Since static structures solved thus far has provided no conclusive clues in this respect, new studies investigating the dynamic nature of PSII might shed more light on this, which is very relevant to make PSII not only a proton pump but also the site of O_2_ evolution. Third, more structural information is needed to figure out the localizations and functions of PsbR and PsbS, PSII subunits that are essential for oxygen-evolving activity (Allahverdiyeva et al., 2007) and photoprotection of plants (Fan et al., 2015), respectively. Last, new high-resolution structures of the photosystems from cyanobacteria, algae, and plants will provide more insights into the evolution of oxygenic photosynthesis, based on which better artificial photosynthetic machineries could be developed.

## Author Contributions

JG wrote the manuscript with the help of HW and QY. YF reviewed and revised the manuscript.

## Conflict of Interest Statement

The authors declare that the research was conducted in the absence of any commercial or financial relationships that could be construed as a potential conflict of interest.
